# Prepulse Inhibition of Acoustic Startle Reflex as a Function of the Frequency Difference between Prepulse and Background Sounds in Mice

**DOI:** 10.1371/journal.pone.0045123

**Published:** 2012-09-11

**Authors:** Sidhesh Basavaraj, Jun Yan

**Affiliations:** Department of Physiology and Pharmacology, Hotchkiss Brain Institute, University of Calgary, Faculty of Medicine, Calgary, Alberta, Canada; University of Salamanca- Institute for Neuroscience of Castille and Leon and Medical School, Spain

## Abstract

**Background:**

Prepulse inhibition (PPI) depicts the effects of a weak sound preceding strong acoustic stimulus on acoustic startle response (ASR). Previous studies suggest that PPI is influenced by physical parameters of prepulse sound such as intensity and preceding time. The present study characterizes the impact of prepulse tone frequency on PPI.

**Methods:**

Seven female C57BL mice were used in the present study. ASR was induced by a 100 dB SPL white noise burst. After assessing the effect of background sounds (white noise and pure tones) on ASR, PPI was tested by using prepulse pure tones with the background tone of either 10 or 18 kHz. The inhibitory effect was assessed by measuring and analyzing the changes in the first peak-to-peak magnitude, root mean square value, duration and latency of the ASR as the function of frequency difference between prepulse and background tones.

**Results:**

Our data showed that ASR magnitude with pure tone background varied with tone frequency and was smaller than that with white noise background. Prepulse tone systematically reduced ASR as the function of the difference in frequency between prepulse and background tone. The 0.5 kHz difference appeared to be a prerequisite for inducing substantial ASR inhibition. The frequency dependence of PPI was similar under either a 10 or 18 kHz background tone.

**Conclusion:**

PPI is sensitive to frequency information of the prepulse sound. However, the critical factor is not tone frequency itself, but the frequency difference between the prepulse and background tones.

## Introduction

Swift reaction to an unexpected sensory stimulus is exhibited in various species of animals including humans [1]–[3]. The reaction is an innate reflexive response based on neural circuits in the lower brainstem [2], [4], [5]. The acoustic startle reflex (ASR) is manifested by a freezing-like movement of the entire body or the constriction of skeleton muscles in response to a sudden and loud sound [1], [6]. The ASR is subject to the modulation of additional sensory stimulus, an important neurological phenomenon. Apart from the influence of sensory signals in the environment on ASR, a weak auditory, visual or somatosensory signal shortly preceding the startle sound can remarkably attenuate the startle response [7]–[12]. This so-called prepulse inhibition (PPI) functions as a sensorimotor gating mechanism that allows the brain to exclude or suppress unnecessary or unrelated sensory inputs [13], [15].

The modulation of the ASR by background or preceding sounds has been extensively studied, particularly in temporal and amplitude domains. In comparison to a silent acoustic environment, continuous broadband noise increases and pulsed broadband noise decreases ASR magnitude [7], [16]. The modulation of ASR by background sound appears to occur within the auditory system since a background visual signal, such as continuous light, has no impact [16]. More profound modulation of the ASR is observed by pulsed sound prior to the startle sound. A preceding noise burst can attenuate ASR magnitude by up to 80% [17]. The prepulse parameters, such as intensity and preceding interval, are important for the modulation of ASR [18]–[20]. When the prepulse sound is less than 500 ms prior to the startle sound, the ASR magnitude decreases (i.e. PPI). Conversely, the ASR magnitude increases when the prepulse sound precedes the startle sound by longer than 500 ms (prepulse facilitation) [21]. The optimal PPI is often observed when the prepulse sound occurs 80–120 ms prior to the startle sound [20]–[22]. In a given preceding interval, longer duration of prepulses could lead to larger decrease in ASR [23], [24]. Similarly, PPI is positively correlated with the difference in intensity between prepulse and background sounds [25]. These results suggest that the ASR magnitude varies depending on acoustic environment, whereas PPI is tightly correlated to the intensity, duration and preceding time of prepulse sound.

Comparatively, the impact of sound frequency on ASR is well studied. The ASR magnitude is smaller when a pure tone rather than broadband noise is used as background sound [26]. It has been shown that high-frequency background noise (8–16 kHz) reduces startle responses regardless of the frequency components of startle sound [26], [27]. Similarly, high frequency background tone reduces the startle responses induced by white noise startle stimulus. On the other hand, low-frequency background noise (1–2 kHz) reduces ASR when the startle stimulus is a low-frequency sound whereas it increases ASR when the startle stimulus is a high frequency sound [27]. The impact of sound frequency on PPI however, remains poorly understood. A recent study demonstrated an inhibitory effect on ASR, i.e., PPI only when the frequency of prepulse tone is lower than background tone [28]. Both ASR and PPI appear susceptive to specific frequency information of prepulse and background sounds.

The present study addresses the impact of frequency differences between prepulse and background tones on PPI. We will demonstrate that a frequency difference of 0.5 kHz appears critical as the PPI systematically enhances below 0.5 kHz and reaches a plateau (∼67%) at 0.5 kHz or higher. Additionally, we will show that the frequency dependence of PPI is similar in 10 kHz and 18 kHz frequency channels.

## Materials and Methods

### Animals

Seven female C57 mice (Charles River Laboratories) aged 7–8 weeks and weighing 16 – 20 g were used in this study. Audiogram analysis and testing for frequency specificity of PPI and ASR were conducted on all seven animals. All protocols and procedures were in accordance with the Canadian Council on Animal Care and the Animal Care Committee, University of Calgary. Animals were allowed to acclimatize to a soundproof room for ∼1 week before testing. They were maintained on a 12-hour light/dark cycle with food and water ad libitum.

### Surgical Procedures

Animals were anesthetized by intraperitoneal injection with a mixture of Ketamine (85 mg/kg) and Xylazine (15 mg/kg). The mouse's head was fixed in a custom-made head holder by rigidly clamping between the palate and nasal/frontal bones. Once the head was positioned, subcutaneous tissue and muscle were removed to expose the area posterior to lambda. A heating pad maintained the animal's temperature within 37–38°C. Anesthetic status of the animal was examined approximately every 40 min by pinching the animal's tail. If the animal showed any responses such as body or tail movement, additional doses of ketamine (17 mg/kg) and Xylazine (3 mg/kg) were injected to maintain the anesthetic level.

### Acoustic Stimulation for Auditory Brainstem Response

Tone bursts were generated by a Real-Time Processor, and were controlled by Brainware software (RP2.1, Tucker-Davis Tech., FL, USA). The tone bursts were delivered from a magnetic speaker placed 45° lateral to and 10 cm away from the mouse's right ear. The speaker was calibrated at the position of mouse's right ear with a condenser microphone (Larson-Davis Model 2520, Dalimar Instruments, Vaudreuil-Dorin, Quebec, Canada). Tone bursts were 60 ms in duration and 5 ms in rise/fall time. Their output ranged from 4 kHz to 30.38 kHz, within an interval of 1.5 octaves (4, 6, 9, 13.5, 20.25, and 30.38 kHz). Sound intensity was expressed as decibel sound pressure level (dB SPL), varied in 10 dB steps from 80 dB SPL to 10 dB SPL, and controlled by a digital attenuator (PA5, Tucker-Davis Tech., FL, USA). The auditory brainstem responses were measured using a frequency/amplitude scan.

### Acoustic Stimulation for ASR and PPI

Two acoustic stimuli delivered through separate speakers were used to test the ASR: a startle stimulus and a continuous background tone. Both acoustic stimuli were generated by RP2.1 and controlled through Brainware/Matlab software. The startle stimulus from RP2.1 was fed in to an audio amplifier and then delivered through a speaker placed 13 cm away and lateral to the animal housing which was constructed of Pyrex glass and brass rods. The continuous background tones were delivered from a magnetic speaker placed 45° lateral to and 18 cm away from the animal's housing. Both speakers were calibrated at the center of the housing using a condenser microphone (Larson-Davis Model 2520, Dalimar Instruments, Vaudreuil-Dorin, Quebec, Canada). The startle stimulus consisted of a white noise burst with duration of 40 ms, and 1ms rise/fall time at 100 dB SPL. Continuous background tones were maintained at 70 dB SPL. In the background dependent ASR experiment, the frequency of continuous background tones consisted of one of the following: white noise, 6, 10, 12, 18, and 26 kHz. In the PPI experiments, 10 and 18 kHz continuous background tones were used. The prepulse stimulus was also a pure tone with 1 ms in rise/fall time and 80 ms in duration delivered at the same intensity as the background tone. The onset of the prepulse preceded the startle sound by 80 ms. The frequency of the prepulse tone was set at 0, 50, 100, 250, 500, 1000, and 2000 Hz below or above the background frequency. Background tone continued following the prepulse tone until the prepulse of the next trial. The trials were randomly separated using an inter-trial interval ranging from 10 to 20 s.

### Measuring hearing sensitivity by auditory brainstem response

Two silver electrodes were used to record response signals. A primary or active electrode was inserted into a 0.5 mm diameter hole drilled at the vertex, 1 mm posterior to lambda. The active electrode was positioned so that it rested on the dura matter. The reference electrode was inserted subcutaneously below the pinna of the right ear. Bioelectrical signals led by silver electrodes were amplified 1000 times and filtered with a bandpass of 100–3000 Hz using an AC Preamplifier (General Purpose AC Preamplifier, Model P55, Grass Technologies). The output from the AC Preamplifier was fed into a RP2.1 real-time processor (Tucker-Davis Tech., FL, USA). Signals of 200 ms after tone onset were digitized and recorded. The auditory brainstem responses to identical stimuli were averaged over 50 trials. The lowest tone intensity capable of evoking a discernable Wave V, an important feature of an ABR waveform, was determined to be the threshold for a particular frequency.

### Measuring ASR

The startle reflex test was carried out with subjects placed in a small custom-made animal housing. The housing was mounted on a plexi-glass base resting on four sensitive piezoelectric transducers (Piezo buzzer, 3–20 VDC, 10 mA at 12V, 2700 Hz buzzer tone), with parallel connections. The piezoelectric transducers converted animal movements into voltage signals. The output of the piezoelectric transducers was converted from analog to digital signals using a Real-time Processor RP2.1 (TDT). The animal movement during the period from 180 ms before to 420 ms after the onset of startle sound was sampled at a rate of 25 kHz. The output signals from the piezoelectric transducers were amplified 100 times, filtered by a bandpass of 25–50 Hz and recorded using BrainWare software (TDT). The recorded data were next processed offline using Matlab software [29]. A webcam was additionally used for observing animal behavior.

### ASR background based experiments

Sessions were divided into three segments. At the start of each session, the mouse was placed in the housing and allowed to acclimate to the environment for ∼5 min. This acclimatization period was followed by the first segment (control) in which the startle sound was delivered without any continuous background sound. In the second segment, the startle sound was delivered with continuous background sounds that were either white noise or a pure tone of 6, 10, 12, 18, or 26 kHz (number of trials ∼10) following 15 s of exposure. The third segment was identical to the first segment in order to examine habituation over the full course of the session.

### PPI based experiments

Sessions were again divided into three segments. The mouse was allowed to acclimate to a continuous background tone for ∼5 min. The acclimation period was followed by an initial startle segment, a second startle with prepulse (PPI) segment and a third startle segment for checking habituation. For the first startle segment, no prepulse was given. In the startle with the prepulse segment, 13 prepulse tones with various frequencies were delivered. The frequency of 1 tone was identical to the frequency of background tone, while 6 tones were lower than and 6 tones were higher than the frequency of the background tone. All prepulse tones were randomly delivered 3 times for a total of 39 trials per PPI session. Background frequencies were either 10 or 18 kHz.

### Data processing

Webcam recordings were used to edit data generated from trials in which the animal showed spontaneous movement. The ASR for each trial was defined as the response to a startle stimulus. The response was assessed for the following parameters: First Peak to Peak Amplitude (ASR_P–P_), RMS Value of ASR (ASR_RMS_), Latency (ASR_LAT_), and Duration (ASR_DUR_).

With regards to the background based ASR experiments, the ASRs were averaged across startle only segments and each class of continuous background tones. The mean amplitude was then calculated for each class of continuous background tones. The means of continuous background tones segment ASR_P–P_ values were normalized to the mean of startle only ASR_P–P_ values. Data were expressed as normalized mean ± standard deviation.

Similarly for the PPI experiments, the ASRs parameters were averaged across startle only trials and each condition of prepulses for both 10 and 18 kHz background frequencies. The mean percent change was then calculated for each prepulse frequency difference with respect to startle only trial mean. Change in percentage was expressed for each ASR parameter using the formula: 100* (1- (Mean of startle with prepulse – Mean of startle only (no prepulse))/Mean of startle only (no prepulse)). Data were expressed as percentage change mean ± standard deviation.

### Statistics

A repeated measures analysis of variance was used to compare the means of three events: 1) auditory brainstem response, 2) ASR to different backgrounds, and 3) percentage of inhibition to varying prepulse frequency within each background frequency. Finally, a repeated measure two-factors analysis of variance was used to asses the means of percentage of inhibition, with prepulse frequency as a within-subject factor and different background frequencies as a subject factor. The data was checked for spherificity using Mauchly's test. If the assumption failed, the Greenhouse-Geisser correction was then applied. A value of *p*<0.05 was considered statistically significant. Furthermore, *post-hoc* tests were done with Bonferroni adjustment to compare within subject significance analysis. All analysis was conducted using SPSStatisitics (IBM) statistical software.

## Results

### Audiogram

**Figure 1:**
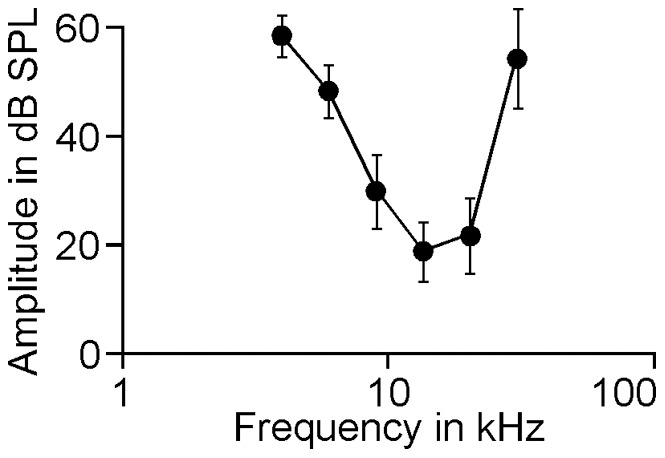
The threshold of auditory brainstem response as a function of tone frequency. The thresholds were significantly lower in responses to 9 kHz, 13.5 kHz and 20.25 kHz than in responses to 4 kHz, 6 kHz and 30.8 kHz (p<0.001). The difference in the thresholds for 9 kHz and 20.25 kHz tones were statistically insignificant, indicating similar hearing sensitivity between the two frequencies.

Firstly, we tested the hearing of all mice by measuring their auditory brainstem responses to frequencies ranging from 4.00 kHz to 30.38 kHz with an interval of 1.5 octaves. As shown in Figure 1, the response threshold varied in response to different frequencies. On average, animals demonstrated low hearing thresholds in a frequency range of 9.00 kHz to 20.25 kHz. Our data suggest the hearing range of animals used in the present study was similar to those previously reported [30], [31].

### Effect of background tone frequency on ASR

**Figure 2:**
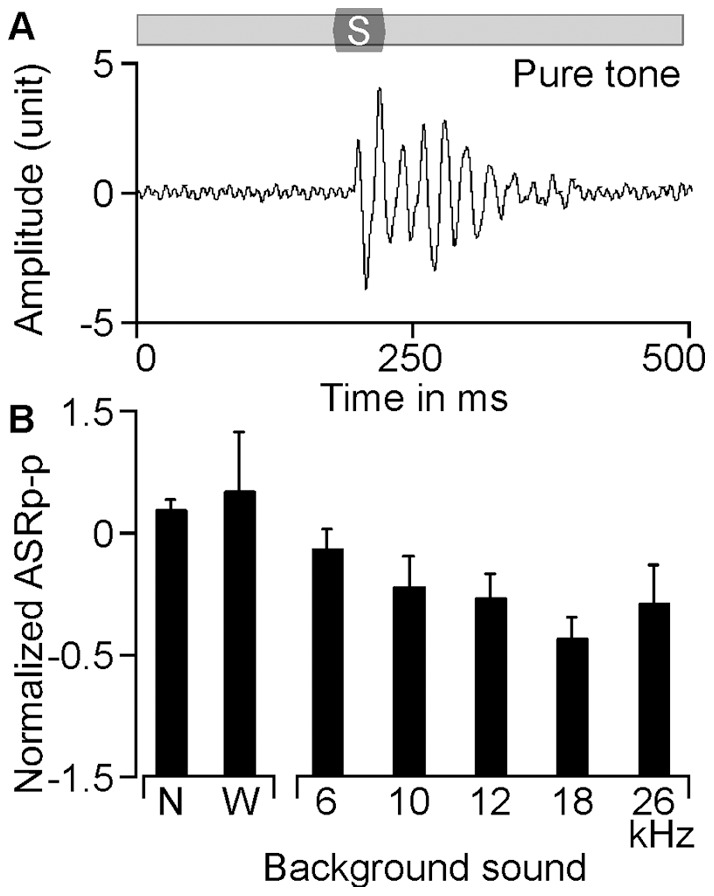
ASR with different background sounds. An example of ASR elicited with a 100 dB SPL white noise burst in the presence of a continuous background tone of 18 kHz at 70 dB SPL (A). Normalized ASR in the presence of no background, white noise, 6 kHz, 10 kHz, 12 kHz, 18 kHz, and 26 kHz (B). ASR was significant smaller for frequency 10 kHz and above than that with white noise background. No significant difference in ASR was demonstrated in the range from 10–26 kHz background frequencies. N: no background sound; S: startle stimulus; W: white noise.

In all C57 mice, ASR was consistently induced with or without background sound. A typical ASR wave

form is shown in Figure 2A. However, the evoked ASR magnitude varied depending on the acoustic background. As seen in Figure 2B, the ASR magnitude in response to white noise was slightly but insignificantly higher than that to a silent environment (1.19±0.22 vs. 1.00±0.04, p>0.05). The ASR magnitude associated with the pure tone background varied with the frequency and was always smaller than that using white noise background. When the frequencies of background pure tones were 10–26 kHz, the ASR magnitude was statistically smaller than that with background white noise (F = 8.906, p<0.001) but insignificant between them (F = 1.171, p>0.05). Excluding the 26 kHz background tone, the higher the frequency of background tone, the lower the ASR magnitude became.

### Frequency-dependent PPI with 18 kHz background tone

**Figure 3:**
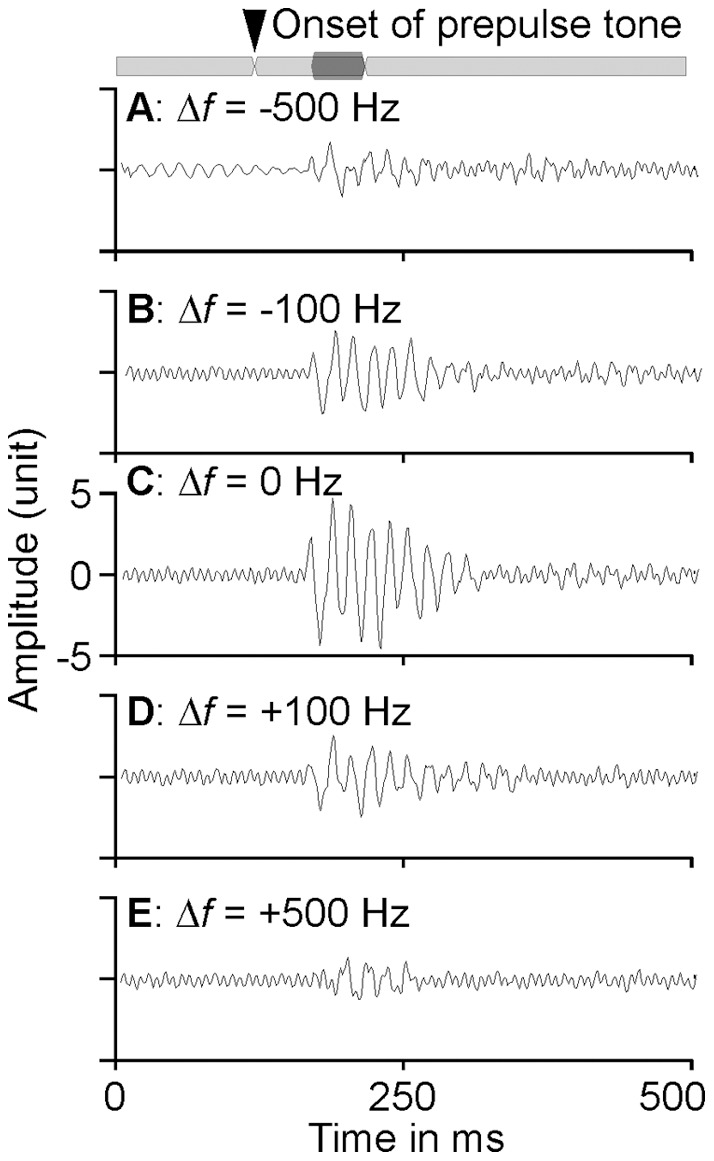
An example of the frequency-dependent prepulse inhibition of the ASR. The background tone frequency was 18 kHz. The frequency of prepulse tone was from 500 Hz higher to 500 Hz lower than the 18 kHz. The ASR magnitude clearly varied with the frequency difference. The light gray bar at the top represents the continuous background tone. The startle is represented by a darkened area, while the prepulse tone is seen between the arrowhead and the offset of the startle sound. The arrowhead represents the onset of the prepulse tone. Δ*f*: the difference of prepulse frequency from the background frequency.

The inhibitory effect of prepulse tone on ASR magnitude is exemplified in Figure 3. The largest ASR was observed when the frequencies of the prepulse and background tones were identical, ie., Δ*f*  = 0 Hz (Fig. 3C). A smaller ASR was observed when the prepulse frequency was either lower, ie., Δ*f* <0 Hz (Fig. 3, A and B) or higher, ie., Δ*f* >0 Hz (Fig. 3, D and E) than the background frequency. In other words, the value of the PPI was influenced by the difference in the frequencies of prepulse and background tones. To show the dependence of PPI on the frequency differences between prepulse and background sound, we examined the percentage change in the first peak-to-peak amplitude (ASR_P–P_), root mean square value (ASR_RMS_), duration (ASR_DUR_) and latency (ASR_LAT_) of the ASR waveform as the function of the frequency differences between prepulse and background tones.

**Figure 4:**
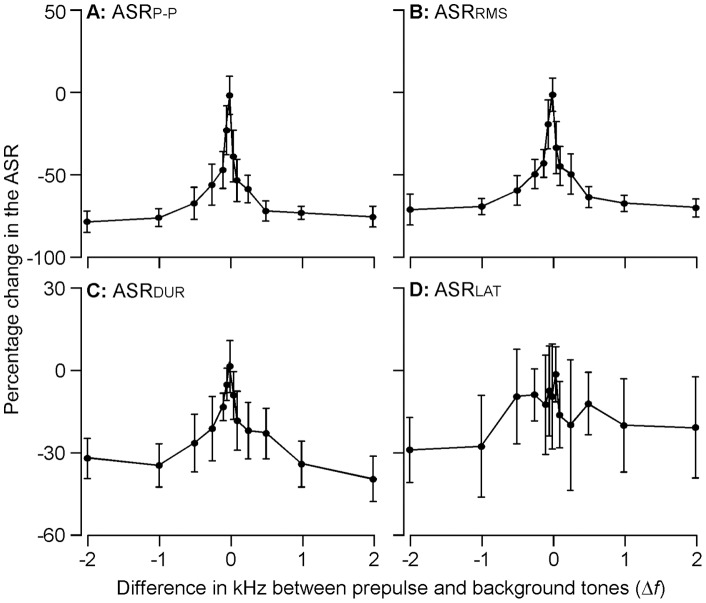
The percentage changes in ASR_P–P_, ASR_RMS_, ASR_DUR_ and ASR_LAT_ as the function of frequency difference (Δ*f*) with a background frequency of 18 kHz. A significant pattern emerges and is associated with the percentage changes in ASR_P–P_, ASR_RMS_, ASR_DUR_ but not for that in ASR_LAT_, i.e., these values systematically decreased as the function of Δf when the Δf was less than 0.5 kHz. A plateau is clearly evident when Δ*f* was at 0.5 kHz and higher.

The ASR_P–P_ systematically decreased with the increase in Δ*f*. As shown in Figure 4A, the A_P–P_ was not inhibited when Δ*f*  = 0. The significant inhibition of ASR_P–P_ was first observed at Δ*f*  = −100 Hz (47.22±12.00%, p<0.01) or Δ*f*  = +50 Hz (39.01±16.84%, p<0.05). It was statistically significant that the inhibition by prepulse with 0<Δ*f* <+0.5 kHz was stronger than that with −0.5<Δ*f* <0 kHz (50.52±10.33% vs. 42.25±16.99, p<0.01). The maximal inhibition was achieved at Δ*f*  = −0.5 or Δ*f*  = 0.5 kHz. The inhibition of the ASR_P–P_ was similar for Δ*f* ≤−0.5 and Δ*f* ≥+0.5 kHz (73.67±1.76%, p>0.05 and 74.07±5.87%, p>0.05).

The percentage inhibition of ASR_RMS_ by prepulse frequency was similar to that of ASR_P–P_ (Fig. 4B). The ASR_RMS_ showed minimal percentage changes when Δ*f*  = 0. Significant decreases in ASR_RMS_ were observed when Δ*f*  = −100 Hz (42.94±9.07%, p<0.001) and Δ*f*  = +50 Hz (33.42±17.13%, p<0.01). The inhibition by the prepulse with 0<Δ*f* <+0.5 kHz was significantly larger than the inhibition by prepulse with −0.5<Δ*f* <0 kHz, ie., ASR_RMS_ decreased by 42.50±8.22% vs. 37.27±15.90% (p<0.001). The maximal inhibition was approached at Δ*f*  = −0.5 and Δ*f*  = +0.5 kHz (59.35±9.9% and 63.64±7.05%). When Δ*f* ≤−0.5 and Δ*f* ≥+0.5, the level of prepulse inhibition was similar (66.59±6.32%, p>0.05 and 67.08±6.28%, p>0.05).

The inhibitory effects of prepulse tone on the ASR_DUR_ appeared to be smaller than those on the ASR_P–P_ and ASR_RMS_ (Fig. 4C). A decrease in ASR_DUR_ by prepulse started to be statistically significant when prepulse frequency was 250 Hz apart from background frequency (30.53±12.79% for Δ*f*  = −250 Hz, p<0.05 and 31.18±11.22% for Δ*f*  = +250 Hz, p<0.05). However, the effect of prepulse tone on ASR_LAT_ showed less correlation to Δ*f* (Fig. 4D).

### Frequency-dependent PPI with 10 kHz background tone

**Figure 5:**
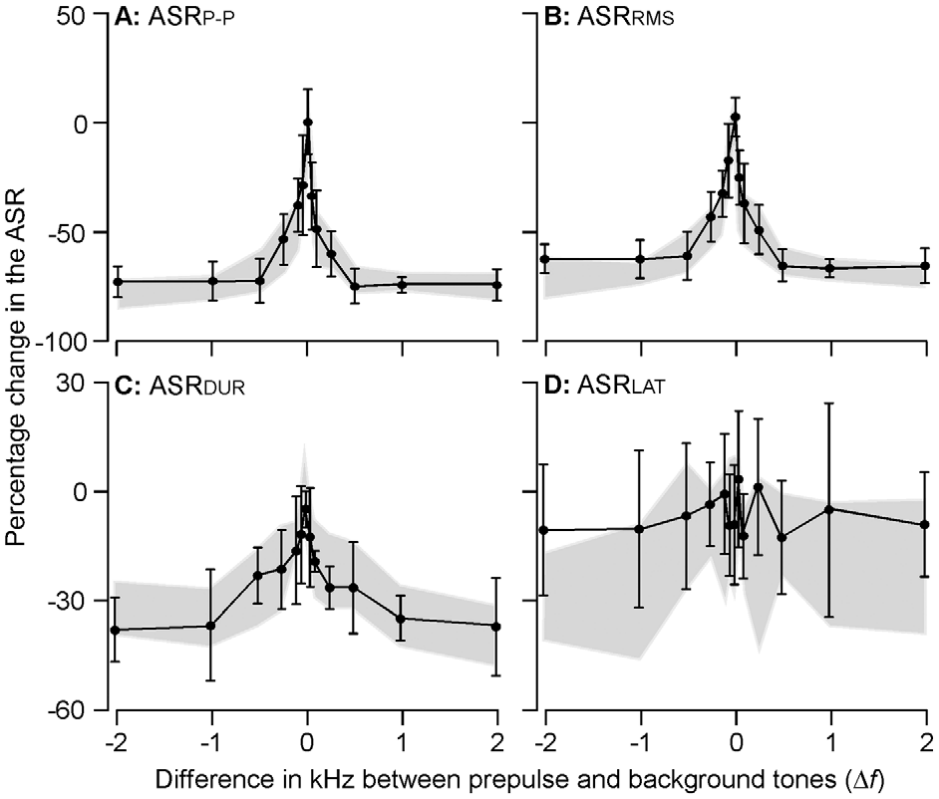
The percentage changes in ASR_P–P_, ASR_RMS_, ASR_DUR_ and A_LAT_ as the function of frequency difference (Δ*f*) when the background frequency was 10 kHz. The pattern is similar to those with an 18 kHz background frequency. The gray areas represent the corresponding areas of the standard deviation in Figure 4.

As shown in Figure 5, the inhibition of ASR by prepulse tone was also dependent on Δ*f* under a 10 kHz background tone.

The prepulse tone with either Δ*f* <0 or Δ*f*>0 reduced ASR_P–P_ as a function of Δ*f* (Fig. 5A). The significant inhibition of ASR_P–P_ was achieved at Δ*f*  = −100 Hz (37.67±13.67%, p<0.05) and Δ*f*  = +50 Hz (33.22±18.88%, p<0.05). Inhibition by prepulse with 0<Δ*f* <+0.5 kHz was stronger than that by prepulse with −0.5<Δ*f* <0 kHz (39.70±12.60% vs. 48.15±13.33, p<0.01). The ASR_P–P_ inhibition reached a plateau at Δ*f* ≤−0.5 and Δ*f* ≥+0.5 kHz (72.48±0.24%, p>0.05 and 74.54±0.31%, p>0.05).

Similar trends were exhibited between prepulse inhibition of ASR_RMS_ and ASR_P–P_ (Fig. 5B). There was little change in ASR_RMS_ when Δ*f*  = 0. The decrease in the ASR_RMS_ by prepulse tone became significant at Δ*f*  = −100 Hz (32.78±11.33%, p<0.05) and Δ*f*  = +50 Hz (25.54±13.33%, p<0.01). The decrease in ASR_RMS_ by prepulse with 0<Δ*f* <+0.5 kHz was significantly larger than that by prepulse with −0.5<Δ*f* <0 kHz (42.50±8.22% vs. 37.27±15.90%, p<0.001). The plateau of the decrease was approached at Δ*f*  = −0.5 and Δ*f*  = +0.5 kHz (59.35±9.9% and 63.64±7.05%). The level of ASR_RMS_ decrease was 66.59±6.32% when Δ*f* ≤−0.5 and 67.08±6.28% when Δ*f* ≥+0.5.

A decrease in ASR_DUR_ by prepulse tone was also correlated to the Δ*f* (Fig. 5C). The decrease became statistically significant at Δ*f*  = −250 Hz and Δ*f*  = +100 Hz (30.83±8.22%, p<0.05 and 28.61±3.05%, p<0.01). The effect of prepulse tone on ASR_LAT_ showed less correlation to Δ*f* (Fig. 5D).

### Comparison of the prepulse inhibition between 10 and 18 background frequencies

To illustrate the potential impact of different frequency channels, we specifically compared the decreases in ASR_P–P_ and ASR_RMS_ by using a prepulse tone under a background tone of either 10 kHz or 18 kHz. The comparison focused on the decreases demonstrated at 0>Δ*f* >−0.5 kHz, 0<Δ*f*<+0.5 kHz, Δ*f* ≤−0.5 kHz and Δ*f* ≥+0.5 kHz. We found no significant differences in both ASR_P–P_ and ASR_RMS_ at any of the above Δ*f* ranges between 10 and 18 kHz background tones. The insignificant differences between the two background frequencies were also shown in the comparison of entire curves between Figures 4A and 5A and between Figures 4B and 5B. The statistical results was F = 0.09 (p>0.05) for the ASR_P–P_ and F = 1.784 (p>0.05) for ASR_RMS_. Similarly, there were no differences for ASR_DUR_ and ASR_LAT_ as seen between Figures 4C and 5C and between Figures 4D and 5D. The statistical results was F = 0.796 (p>0.05) for ASR_DUR_ and F = 6.685 (p>0.05).

## Discussion

The neural circuit for the ASR is relatively simple. The startle sound is transmitted from the cochlear nucleus directly to the caudal pontine reticular nucleus and indirectly via the nucleus of the lateral lemniscus [2], [4], [5]. The caudal pontine reticular nucleus directly projects to premotor or motor neurons in the brainstem and spinal cord [4]. The neural substrates for the PPI are more complex, involving many neural structures in higher level sensory systems and in the limbic system [13], [14], [32]. A common route for modulating PPI is the innervation of the pedunculopontine tegmental nucleus by the inferior colliculus, superior colliculus and higher nuclei. The pedunclopontine tegmental nucleus then activates the caudal pontine reticular nucleus [32]. This reflex arc for the PPI can functionally be divided into three parts: the central auditory system, limbic system, and motor system. A primary task of the central auditory system is the detection of novel acoustic signals, ie., the differentiation of an acoustic signal from acoustic background [33]. Auditory information then simply passes on to the second part, the limbic system [34], [35]. The limbic system is mostly involved in cognitive information processing [36], [37]. Nuclei in the limbic system in turn modulate the activity of the motor system, the third part through the caudal pontine reticular nucleus [35]. The dissimilar functionalities of the first two systems (central auditory and limbic systems) suggest that some properties such as frequency-dependence of PPI are related to the physical parameters of the prepulse signal whereas others such as sensorimotor gating, are not.

In the present study, both prepulse and acoustic backgrounds were pure tones. The only difference between them was tone frequency. We demonstrate that the PPI was highly dependent on the disparity of the frequencies between prepulse and background tones. When the difference in frequency was less than 0.5 kHz, the inhibition of startle response by prepulse tone was enhanced as the function of the frequency difference. The inhibition reached a plateau when the frequency difference was 0.5 kHz or larger (Figs. 4 & 5). This was best shown by ASR_P–P_ and ASR_RMS_ (Figs. 4A–B & 5A–B), suggesting that this 0.5 kHz difference is critical for the mouse auditory system to competently discriminate the prepulse tone from the background tone. In other words, the spectral difference allows the auditory system to comfortably detect a novel signal from the background sound. Our findings are conceptually in line with previous observations on the impact of preceding sound intensity and duration. Higher intensity and longer duration of prepulse sound typically produce stronger inhibition of the startle responses [18], [20]. These findings suggest that the PPI is dependent on the parameters of prepulse sound.

One issue to be clarified here is the biological or neurological significance of this sound-parameter dependence. The relationship makes sense in terms of our understanding of the first part of the PPI neural circuit but less obviously so with the second part. The exact information sent from the sensory system to the limbic system remains vague, but it is clear that the limbic system processes cognitive information instead of sensory information [36], [37]. Numerous studies have demonstrated that startle responses can be inhibited by prepulse sound, light and touch [7], [8]. Even when the prepulse is an acoustic signal, either white noise or pure tone is able to suppress the startle response with or without background sound [20]. That there is no difference in the inhibition of the startle response by either pulsed or continuous preceding sound has also been demonstrated [38]. Furthermore, when a background sound is presented, a preceding silence or gap also produces the PPI [39]. In this case, the PPI shows an insignificant correlation to the shift in the frequency of background sound [39], [40]. These findings strongly suggest that the PPI does not depend on the physical properties of the prepulse signal, including sound frequency and amplitude. The novelty and/or context carried by the preceding signal appear to be the critical factor [38], [41]. This was analyzed in the present study by conducting PPI experiments with two different frequency channels (10 kHz and 18 kHz channels). Figure 5 clearly shows that the functional curves and deviations of ASR_P–P_, ASR_RMS_ and ASR_DUR_ vs. Δf were well matched between 10 kHz (lines in Fig. 5, A–C) and 18 kHz (gray areas in Fig. 5, A–C) background tones. These data suggest that the PPI was determined by the frequency difference (transient state) between prepulse and background tones but unrelated to the frequency (steady state) of background tone. The frequency dependence of the PPI reflects the capacity for signal processing or sound discrimination in the auditory system.

Due to the ease of operation and predictable features of the prepulse signal across species, the PPI can be an ideal measure of sensory function (1st part of the reflex arc), cognitive function (2nd part) and motor function (3rd part) with appropriate operant procedures. The assessment of cognitive disorders with an abnormality in sensory motor gating function by the PPI is well documented. This application is typically exemplified in the schizophrenia that is featured with impaired inhibition or control of incoming sensory information, ie., so called sensory information “flood.” Deficits in PPI are reliably identified in schizophrenic patients and animal models [42], [43]. Besides PPI involvement, the roles of other properties of the prepulse sensory signal have also been confirmed. For example, this study and others demonstrate the correlation of PPI with the frequency and intensity of prepulse sound [20], [28], [40], [44]. Our findings promote us that the PPI can be a valuable approach in assessing the function of sensory modality.

Frequency discrimination is a fundamental and vital feature of the auditory system. Any deficit in frequency discrimination correlates to hearing abnormality including language learning and development skills [45]. The assessment of frequency discrimination is generally easier in human subjects. Performing two-tone frequency discrimination in animals typically requires extensive training or learning before the testing [46]. Outcomes are potentially impacted however, by the learning abilities of animals as well as by the effectiveness of devised training methods. Our data showed detailed frequency dependence of the PPI and suggest that the PPI can be an efficient paradigm for assessing animal discrimination of any two frequencies with a resolution of up to 0.5 kHz. Although the ASR magnitude varies with the frequency of background tone in our study (Fig. 4) and others, PPI measured in percentage change is independent from the amplitude of control ASR [40]. There are two obvious procedural advantages in assessing frequency discrimination using this method. No prior training is required before testing and the testing can be completed in a shorter time-frame. In addition, the PPI is also useful for assessing animal learning ability [47], [48]. Setting the frequency difference between prepulse and background tone at less than 0.5 kHz, allows us to assess changes in the PPI after learning. Similarly, this paradigm also allows examining the negative impact on frequency discrimination due to drug effects, sensorineural hearing loss and neurological disorders [45], [47], [48] by setting the frequency difference at slightly higher than 0.5 kHz.

It is necessary to note here that the ASR_LAT_ showed poor correlation with Δf under both 10 kHz and 18 kHz background tones (Figs. 4D & 5D). The standard deviations at each Δf were also different between 10 kHz (solid lines in Fig. 5D) and 18 kHz (gray shaded area in Fig. 5D). These results are likely due to variations within individual samples and should be clarified. At present, this parameter cannot be reliably used to measure frequency discrimination.
